# From Oppression to Violence: The Role of Oppression, Radicalism, Identity, and Cultural Intelligence in Violent Disinhibition

**DOI:** 10.3389/fpsyg.2018.01505

**Published:** 2018-08-20

**Authors:** Roberto M. Lobato, Miguel Moya, Manuel Moyano, Humberto M. Trujillo

**Affiliations:** ^1^Department of Social Psychology, Faculty of Psychology, University of Granada, Granada, Spain; ^2^Department of Psychology, Faculty of Education Sciences, University of Cordoba, Cordoba, Spain; ^3^Department of Behavioral Sciences Methodology, Faculty of Psychology, University of Granada, Granada, Spain

**Keywords:** cultural identity, cultural intelligence, perceived oppression, radicalism, violent disinhibition

## Abstract

Violent radicalization and terrorism continue to pose social and security problems. Starting from the theoretical framework offered by the significance quest theory, the purpose of this research was to analyze the different roles that radical intentions play in the relationship between the loss of significance and violent disinhibition in Muslims and non-Muslims. For this reason, we carried out two studies: the first one with 133 Muslims and 126 non-Muslims, and the second with 98 Muslims and 167 non-Muslims. Specifically, we measured how perceived oppression influenced violent disinhibition through radical intentions. Secondly, we also measured the impact of identity and cultural intelligence in these relations. The main finding of the research was that there was an indirect effect of perceived oppression on violent disinhibition through radical intentions in the Muslim sample, whereas, in the non-Muslim sample, the effect of perceived oppression on violent disinhibition was not mediated by radical intentions. These results were replicated in both studies. Additionally, we found that identity and culture were factors that moderated the proposed relations. This work therefore shows that the conjunction of the loss of significance and radical intentions seems to strongly exacerbate the likelihood of a process of violent disinhibition for those who are considered to be in marginal contexts. Overall, different pathways and intervening factors are in the process of radicalizing Muslims and non-Muslims in Western societies.

## Introduction

For some time now, jihadist radicalization has been receiving preferential attention from social media, as well as though the political agenda of the European Union. Likewise, terrorism on European soil derived from radicalism has become one of the greatest security challenges facing Western countries. Taking this into consideration, the study of these topics is crucial to support evidence-based decision-making. It is necessary for all academic fields, including psychology, to use all their theoretical, empirical, and methodological resources, both basic and applied, to deal with radicalization and terrorism. This social problem, currently aggravated by the return of foreign terrorist fighters from Syria and Iraq after the fall of the so-called Islamic State (Barrett, 2017; Cragin, 2017), has given rise to different theoretical perspectives and empirical investigations that have tried to explain the process of violent radicalization, as well as the factors that contribute to it (Trujillo et al., 2006b; Dugas and Kruglanski, 2014; Gómez et al., 2017; Trujillo and Moyano, 2018). Consequently, this work intends to review previous studies and delve into the factors that underline radicalization processes and their relationships with cultural and religious identities.

## The Process Of Radicalization

Radicalization can be defined as a psychosocial process that entails an increase in the commitment to some kind of extremist ideology (Horgan, 2009). In the last decade, different theories and models have been developed to explain this complex and multivariate process. Among them, the significance quest theory (Kruglanski et al., 2009a, 2013, 2014, 2017) has obtained outstanding empirical and experimental evidence that has allowed it to reinforce some of its postulates and develop proposals for preventive intervention in the community and in risk management (Bélanger, 2017).

Basically, the significance quest refers to the people’s need “to make a difference,” “to be important,” and “to be someone.” According to Kruglanski et al. (2009a, 2014) there are three events that can trigger the quest for significance: (1) the loss of significance (e.g., humiliation, social alienation); (2) the perception of threat to the significance (e.g., the possibility of being rejected); and (3) the opportunity to gain considerable significance (e.g., becoming a hero, a martyr). The significance quest theory suggests that the (potential) loss of significance and the opportunity to gain it stimulate collectivistic goals to restore the loss of significance, which in turn motivates the defense on behalf of the group (Dugas et al., 2016). This perspective suggests that the quest for personal significance may encourage radicalization under certain conditions. For the radicalization process to take place, three elements are necessary (Kruglanski et al., 2014): (1) the quest for significance to be activated; (2) violence to be identified as a means to achieve significance; and (3) the commitment to use violence to achieve significance and become dominant and incompatible with other possible sources of significance. Considering these three elements, opting for violence as a means to achieve significance to the detriment of more prosocial means will depend, to a large extent, on different variables such as the ideology of the group (Kruglanski et al., 2014) or the need for cognitive closure (Kruglanski et al., 2009b; Webber et al., 2018).

In a complementary way, the psychosocial model of recruitment and violent mobilization (Trujillo et al., 2006a,b; Trujillo and Moyano, 2018) has developed theoretically and empirically developed how the recruitment of an individual occurs within a violent group. According to this model, the recruiters enlist and guide the new recruits through the following phases: (1) identification of the individual in critical environments (marginal scenarios); (2) uptake of the individual in mental imbalance (first approach to the potential recruit); (3) psychological subjection and the consequent psychological alienation; (4) ideological indoctrination of a political and religious nature (doctrinal alienation); (5) violent disinhibition through the application of strategies aimed at legitimizing violence; (6) training for the exercise of violence; and (7) logistical support for the execution of violent actions.

Thus, in order for someone, after being indoctrinated, to go one step further and become a violent extremist, the individual must firmly believe in the legitimacy of violence. For this, recruiters often use strategies based on narratives that legitimize such violence. It is worth highlighting that, among other possible narratives, recruiters often use ones that (1) extol the mistreatment unjustly suffered by an indoctrinated person; (2) look for and clearly identify those guilty of mistreatment, who will be the victims of the violent actions; (3) attribute the guilt of all the ills suffered to the potential victims, who are not only clearly guilty but also deserving of the greatest punishment; (4) dehumanize victims through the use of adjectives that reify or animalize them; (5) displace the responsibility to exercise violence toward a superior and unquestionable being (i.e., God); (6) justify violence based on superior principles (moral values, symbols, norms, roles, etc.) that transcend the indoctrinated ones themselves; (7) disperse the responsibility to exercise violence to everyone; and (8) extol violence as the only instrument for the indoctrinated to recover from the mistreatment suffered. These narratives show revenge as the best instrument to achieve a new life that is more dignified and shows more sense and certainty for all.

## From Oppression to Violence

For a premeditated and intentional action classified as violent to be triggered, there must be a disinhibition that allows it beforehand. Violent disinhibition refers to the desire to end one’s own life or that of others, a high negative emotionality (hostility, dislike, aversion, hatred, anger, and tension), and the observational learning of violent behavior (Trujillo, 2009; Moyano and Trujillo, 2013, 2014; Trujillo and Moyano, 2018). Likewise, for a subject to act decisively to commit terrorist acts, usually there is a prior event or circumstance that precipitates the radicalization process (e.g., a grievance).

Among the triggers of the quest for significance, two stand out: humiliation directed at the in-group and humiliation directed at personal circumstances (Webber et al., 2018). Both circumstances can generate a loss of significance, which could be enough to awaken the quest (Kruglanski et al., 2013). On the other hand, from the point of view of the psychosocial model of recruitment, perceived or real marginality is one of the features that could increase the risk of recruitment (Moyano and Trujillo, 2016). Both marginality and humiliation are variables potentially linked to the oppression construct, another factor that has been considered a potential contributor to the process of radicalization (Victoroff, 2005; Victoroff, 2009, Unpublished). Basically, oppression is understood as the perception of subjugation of one group by another, imposed by an asymmetric power and often reinforced by hostile conditions such as threats or actual violence. However, although oppression is an important predecessor of violent disinhibition (Berkowitz, 1989), it does not appear to be its necessary or sufficient cause (Victoroff, 2009, Unpublished).

Therefore, it is plausible that, in the functional relationship between both variables, oppression and violent disinhibition, there is another variable (or variables) that mediates this relationship. Following the phases of the psychosocial model of recruitment previously exposed, after identification and recruitment, an ideological indoctrination would be necessary (Trujillo and Moyano, 2018), that is, a totalitarian ideology that unites the subject to the group and legitimizes the use of violence in its defense. Such a variable would constitute a stage prior to violent disinhibition. A construct that satisfies these conditions are the radical intentions, defined as the intention to engage in illegal and violent acts in defense of the in-group (Moskalenko and McCauley, 2009). Although radicalism could be a mediator of this relationship, two key points must be considered: the first is that radicalism does not always lead to violence (Bartlett and Miller, 2012); and the second is that it is foreseeable there are cultural differences in the process of radicalization.

## The Role of Identity and Culture

The evidence has also shown that identity and cultural factors could play an important role in the process of radicalization. Thus, Lyons-Padilla et al. (2015) found that the more discrimination experienced by Muslim Americans, the less purpose and meaning they had, especially in the case of those perceived without a cultural referent. In other words, marginalization did not predict support for more fundamentalist groups directly but through the loss of significance. In summary, for immigrants or minority groups who feel they are marginalized and experience higher rates of need for closure, the loss of significance seems to have a strong relation with support for extreme groups or measures. Therefore, it is predictable that in European societies, especially in certain contexts associated with the risk of social exclusion and marginalization (Shavit, 2014), the effect will be stronger for Muslims than for non-Muslims when they suffer a loss of significance.

Other research has analyzed the cultural dependence on the need for cognitive closure. Webber et al. (2018) found in different cultural samples that avoidance of uncertainty, or the need for cognitive closure, alongside the lack or the loss of significance, can lead to adherence to extremist organizations that justify violence. This point is related to the studies of Kosic et al. (2004), who found a relationship between the higher need for closure and bad acculturation. As we can see, the relationships between identity, culture, and radicalization are complex and influenced by different variables.

In that sense, using data from the Pew Research Center surveys on Muslim residents in European and American territories, Victoroff et al. (2012) found that perceived discrimination toward Muslims was associated with the legitimization of suicide terrorism, being a risk factor for political violence. Furthermore, in a field investigation developed in a vulnerable urban environment, Moyano and Trujillo (2014) found a significant relationship between perceived oppression, radical intentions, and violent disinhibition in a Muslim sample, whereas the same relations were not significant in a sample of Christians. They concluded that these results are due to a greater cohesion with religion on the part of the Muslim sample and the context’s demographic characteristics, which include the perception of an out-group threat residing in a marginal neighborhood. Thus, religious or cultural identity seems to be an important factor that strengthens the relationship between the perception of oppression, radical intentions, and violent disinhibition.

Finally, we highlight the role of cultural intelligence (Earley and Ang, 2003; Ang and Van Dyne, 2008). This construct represents the capacity to effectively adapt to contexts with a strong cultural component, and it could be considered a protective factor against radicalization and violent extremism (Fang et al., 2018; Trujillo and Moyano, 2018). Although it is a territory that still needs to be explored, Moyano et al. (2015) found that cultural intelligence showed a negative relationship with constructs such as the meaning of life and the need for cognitive closure, which, in turn, have been proposed as factors influencing violent extremism (Klein and Kruglanski, 2013; Kruglanski et al., 2015; Trujillo et al., 2016). This leads to the assumption that cultural intelligence could also be acting as a moderator in the process of radicalization so that people with lower levels of cultural intelligence would be more willing to use violence.

All the exposed factors represent only some of those that have been associated with the radicalization process. In this work, we try to provide empirical evidence with an exploratory intention that helps to deepen the knowledge of the interrelations between potentially relevant factors and using interesting samples from an ecological point of view. That is, the proposed relations establish that the loss of significance will trigger violent disinhibition. These relations could be enhanced by a violent narrative that supports pro-group radical actions. Given the cultural and identity differences between Muslims and non-Muslims in the context of Western societies, we propose that the radicalization processes will be different for both groups.

## The Present Investigation

Based on the reviewed empirical evidence, it is expected that the perceived oppression will facilitate violent disinhibition, in both Muslim and non-Muslim residents in Spain, and that this relationship will be mediated by the radical intentions in a different way in both groups due to the identity and cultural differences. To verify these assumptions, two studies that seek to answer the following hypotheses have been proposed:

(1) In Muslims, the radical intentions will have a mediating effect on the relationship between perceived oppression, considered a predictor variable, and violent disinhibition, considered a criterion, and there will be no such mediating effect in non-Muslims.

(2) Social identity and cultural intelligence will moderate the indirect effect of mediation previously proposed, in the sense that those with a stronger cultural identity and lower cultural intelligence will be more prone to violent disinhibition. Due to the lack of more concrete evidence, this hypothesis is considered exploratory because it cannot be confirmed what moderating relationships will occur or if differences will arise between Muslims and non-Muslims.

## Study 1

In the first study, it was evaluated whether the radical intentions are mediating the relationship between perceived oppression, considered a predictor variable, and disinhibition to violence, considered a criterion variable. To this end, a sample of Muslims and a sample of non-Muslims who lived in areas considered at risk for radicalization were established.

The samples were collected from two marginal neighborhoods in Spain where Muslims and non-Muslims coexist in similar proportions (Trujillo et al., 2011; Moyano and Trujillo, 2013, 2014; De la Corte, 2015). One of them is the Autonomous City of Melilla, and the other is the neighborhood of El Puche, located in the city of Almería. In both contexts, interventions by the security forces and critical incidents associated with jihadist radicalization have been documented (Reinares and García-Calvo, 2015, 2016).

### Materials and Methods

#### Participants

The sample consisted of 259 young people (133 Muslims and 126 non-Muslims), all of them students in two secondary education institutes. Of the 259 students, 119 attended an institute in the neighborhood of Puche in the city of Almería (city in southeast Spain), and 140 attended an institute in the Autonomous City of Melilla (Spanish city in North Africa). The students of both institutes belonged to a social context with a high risk of social exclusion. With regard to gender, the group of Muslims was composed of 64 men, 66 women, and three unidentified; and that of non-Muslims had 56 men and 70 women. The age of the participants was between 13 and 18 years old (*M* = 15.89, *SD* = 1.05). Regarding the sample, despite our attempts to get a large sample, access to people in these social contexts limited the sample size. On the other hand, according to Fritz and MacKinnon (2007), a sample of 90 participants are required to get an indirect effect of 0.16 given that both paths are estimated to be medium size (0.39) if the Sobel test is performed. Thus, given the circumstances, we believe we have adequate power.

#### Evaluation Instruments

Two types of questionnaires with the same measures were prepared, both in Spanish, but with a variation in the wording for some of them depending on whether they were intended for Muslims or non-Muslims. Muslims evaluated the oppression by non-Muslims and the intentions to defend their own group, while non-Muslims did the same with respect to the group of Muslims.

##### Religious group

The participants responded to an item identifying their religious group (Muslim religion vs. Christian religion).

##### Perceived oppression

A Spanish adaptation of the *Oppression Questionnaire*, an instrument that consists of 32 items that assess perceived oppression (α = 0.95; Victoroff et al., 2006; Moyano, 2011), was applied. The items were scored on a Likert-type scale from 1 (totally disagree) to 4 (totally agree), for example, “My group is often treated unfairly.” In this, the perceived oppression between Muslims and non-Muslims was assessed.

##### Radical intentions

The participants completed the Moskalenko and McCauley (2009) adapted to Spanish by Trujillo et al. (2016; α = 0.90). The scale comprises four items (e.g., “I would continue to support an organization that fights for the political and legal rights of my group even if it sometimes goes beyond the law,” “I would attack the police or the security forces if I saw them hit members of my group”). The evaluation of the items was done by means of a Likert-type scale with scores of 1 (“totally disagree”) to 7 (“totally agree”). Higher scores indicate greater radical intentions.

##### Violent disinhibition

The Disinhibition to Violence subscale (α = 0.82), which forms part of a broader instrument, the Questionnaire on Risk of Islamist Radicalization in Young People (Moyano, 2011), whose objective is to evaluate multiple factors potentially contributing to radicalization, was applied. This subscale is made up of four items that assess violence intentions to the self (“In the last month I have had the desire to end my own life”) or to others (“In the last month I have had wishes to end the lives of others”), hatred toward others (“In the last month I have felt hatred for some people”), and exposure to models that favor violence (“My friends continually talk about fights and violent issues”). The items were scored on a Likert-type scale with scores between 1 (“totally disagree”) and 5 (“totally agree”).

##### Sociodemographic variables

Information on sociodemographic variables such as age and gender was also recorded.

#### Procedure

The participants collaborated on a voluntary basis once the study was approved by the School Council, and a written informed consent was obtained from the participants’ parents. The evaluation took place in the classrooms of the respective centers for approximately 50 min. The centers’ staff collaborated with the application of the evaluation instruments.

### Results

#### Muslims

**FIGURE 1:**
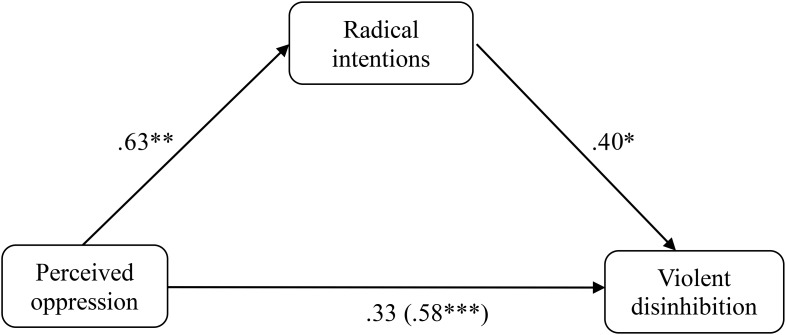
Mediation of oppression on violent disinhibition through the radical intentions in the Muslim sample of Study 1. ^∗^*p* < 0.05; ^∗∗^*p* < 0.01; ^∗∗∗^*p* < 0.001, *typified scores.*

In order to determine whether the radical intentions exert a mediating effect on the relationship between perceived oppression and violent disinhibition in the sample of Muslims (*N* = 113), following the recommendations of Baron and Kenny (1986), and Kenny (2014), different regression analyses were performed (**Figure 1**). The results indicated a statistically significant predictive capacity of perceived oppression on the radical intentions (β = 0.625, *p* < 0.001, *R*^2^ = 0.374), of the radical intentions on violent disinhibition (β = 0.618, *p* < 0.001, *R*^2^ = 0.373), and of the perceived oppression on violent disinhibition (β = 0.576, *p* < 0.001, *R*^2^ = 0.314). After carrying out the three simple regressions described above, a multiple linear regression was performed, taking the perceived oppression and the radical intentions together as predictors and the violent disinhibition as a criterion. The results of the analysis indicated that when the radical intentions predictor is introduced into this multiple regression model, perceived oppression loses its predictive capacity on violent disinhibition (β = 0.325, *p* = 0.051), whereas the radical intentions continues to show predictive capacity on violent disinhibition (β = 0.402, *p* = 0.017). Following the suggestions of Beckstead (2009) and Kenny (2014), the Sobel (1982) test was subsequently performed, and the results showed a significant mediating effect of the radical intentions (*z* = 3.872, *p* < 0.001). Given the possible controversy in the causal relationship between radical intentions and violent disinhibition, the same analysis was realized when changing the mediator variable for the dependent variable; that is, violent disinhibition became the mediator and radical intentions the dependent variable. The Sobel test did not show a significant mediating effect for violent disinhibition (*z* = 0.796, *p* = 0.213).

#### Non-Muslims

**FIGURE 2:**
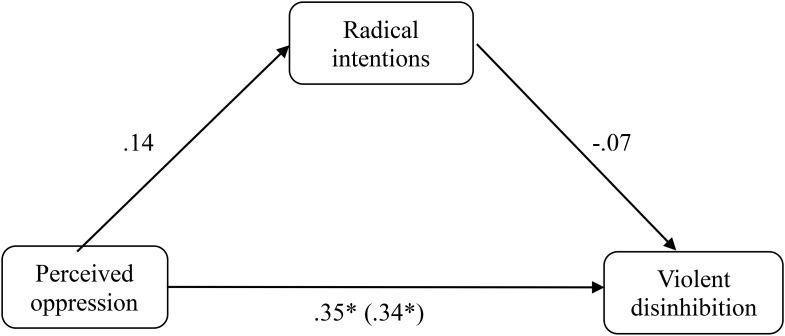
Mediation of oppression on violent disinhibition through the radical intentions in the non-Muslim sample of Study 1. ^∗^*p* < 0.05; ^∗∗^*p* < 0.01; ^∗∗∗^*p* < 0.001, *typified scores*.

In the non-Muslim group (*N* = 126), the same analyses as those indicated above for the group of Muslims were carried out (**Figure 2**). The results indicated that the perceived oppression did not significantly predict the radical intentions (β = 0.136, *p* = 0.410, *R*^2^ = 0.008) and the same is true for the radical intentions on violent disinhibition (β = 0.020, *p* = 0.950, *R*^2^ = 0.023). However, predictive capacity was found for perceived oppression on violent disinhibition (β = 0.344, *p* = 0.037, *R*^2^ = 0.093). Once these three simple regressions were carried out, a multiple linear regression was performed, taking the perceived oppression and the radical intentions together as predictors and the violent disinhibition as a criterion. The results of the analysis indicated that when the radical intentions predictor is introduced in the multiple regression model mentioned above, the perceived oppression maintains its predictive capacity on violent disinhibition (β = 0.353, *p* = 0.036), whereas the radical intentions still shows no predictive capacity on violent disinhibition (β = −0.073, *p* = 0.654). Furthermore, similar to the sample of Muslims, when radical intentions was the dependent variable, the mediation effect of violent disinhibition was not significant (*z* = 684, *p* = 0.304).

### Discussion

In summary, it can be said that the proposed hypothesis was confirmed. In the case of Muslims, the perceived oppression is not a direct predictor of violent disinhibition because the radical intentions have a mediating effect in this functional relationship. Thus, for perceived oppression to generate violent disinhibition in young people of the Muslim religion, it is necessary that they show an attitude toward radicalism. On the other hand, perceived oppression is a direct predictor of violent disinhibition among non-Muslims, since, in this functional relationship, radical intentions do not exert a mediating effect; consequently, for perceived oppression to generate violent disinhibition in young people of the non-Muslim religion, it is not necessary for them to show an intention toward radicalism.

## Study 2

Given the results of Study 1, a second study was conducted to replicate and possibly ratify the results of the first one (Hypothesis 1) and, in addition, to verify if other factors such as cultural identity and cultural intelligence acted as moderating variables of the proposed mediations (Hypothesis 2). For this, moderated mediations were carried out, and, once again, a sample composed of both Muslims and non-Muslims was established.

### Materials and Methods

#### Participants

The sample consisted of 265 participants (98 Muslims and 167 non-Muslims) living in the Spanish city of Granada. The sample was collected at the bus station and in areas adjacent to the mosques and neighborhoods where there is a greater proportion of Muslims. The participants were randomly selected; they were approached in the street, and after understanding and accepting the application of the questionnaire, they were given the necessary time to complete it. The number of participants was intentionally kept balanced in terms of gender. Of these, 174 were Spanish by birth, 16 were children of immigrants with Spanish nationality (second generation immigrants), 19 had dual citizenship, 45 were foreigners residing in Spain, and 11 did not report nationality. Regarding gender, the group of Muslims was composed of 40 men and 58 women, and that of non-Muslims was 70 men and 97 women. The age of the participants was between 18 and 75 (*M* = 28.04, *SD* = 10.60).

Among Muslims, 89.9% declared themselves to be practitioners compared to 10.1% who declared themselves to be non-practicing. In the non-Muslim group, 56.3% considered themselves Christians and 43.7% declared not to have any religion. Of those who considered themselves Christians, 28.1% indicated that they were practitioners, while 71.9% considered themselves non-practicing.

Among Muslims, 17% of the participants never attended a mosque, 37.5% did so on special occasions, 9.1% once a month, 3.4% once every fortnight, 18.2% once a week, 5.7% 3 or 4 days a week, 5.7% every day, and 3.4% several times a day. Regarding the frequency of prayer, 4.5% of the participants never prayed, 3.4% almost never, 3.4% occasionally, 5.6% several times a year, 7.9% often, 1.1% very often, and 74.1% every day. The exclusivity of God, that is, if their God was the only true God, was also inquired, with an 11-point Likert-type scale (0 = not at all agree to 10 = totally agree). The mean was 8.40 (*SD* = 3.22).

In the sample of non-Muslims, 40.7% of participants never attended church, 49.1% did so on special occasions, 4.8% once a month, 0.6% once every fortnight, 4.2% once a week, and 0.6% 3 or 4 days a week. Regarding the frequency of prayer, 46.7% of participants never prayed, 19.3% almost never, 9% occasionally, 7.3% several times a year, 11.5% often, 1.9% very often, and 4.3% every day. The exclusivity of God was also inquired, with an 11-point Likert-type scale (0 = not at all agree to 10 = totally agree). The mean was 1.33 (*SD* = 2.56).

#### Evaluation Instruments

All the participants completed one of the four versions of the questionnaire dealing with the counterbalance of the measures and their religious group (Muslims vs. non-Muslims). No significant differences were found between the two versions regarding the order. Taking into account the variations among Muslims and non-Muslims, the same variations in wording were found in Study 1; that is, Muslims evaluated the oppression by non-Muslims and the intentions to defend their own group, and non-Muslims evaluated the oppression by Muslims and the intentions to defend the in-group. In all of the proposed measures, except for those in which another scale was specified, a 5-point Likert-type response scale was used (completely disagree to completely agree).

##### Group identification

A measure composed of two group identification indicators (Western-Christian and Arab-Muslim culture) was used, applying a 7-point Likert-type scale. Higher scores indicate greater group identification.

##### Perceived oppression

A reduced version of the scale used in Study 1 (R-OQ questionnaire; Lobato, 2017, Unpublished) was used to measure the perceived oppression. In this case, oppression was measured by the cultural out-group (Western-Christian vs. Arab-Muslim) (α = 0.93).

##### Radical intentions

The same scale as in Study 1 was used (α = 0.81).

##### Violent disinhibition

The Disinhibition to Violence subscale used in Study 1 was applied (α = 0.65).

##### Cultural intelligence

Ang et al. (2007) scale adapted to Spanish by Moyano et al. (2015) was used. The scale consists of 20 items that evaluate four factors: (1) the meta-cognitive component (“I am aware of cultural differences and adapt to them in interactions with people from other cultures”; α = 0.72); (2) the cognitive component (“I know the cultural values and religious beliefs of other cultures”; α = 0.87); (3) the motivational component (“I enjoy living in other cultures not familiar to me”; α = 0.83); and (4) the behavioral component (“I alter my facial expression when the cultural interaction situation requires it”; α = 0.84). Higher scores indicate greater cultural intelligence.

##### Sociodemographic variables

Information on sociodemographic variables such as religion, age, and gender was also recorded.

#### Procedure

The participants completed the survey without receiving any compensation. Once they were presented with the survey, they first accepted its terms and then proceeded to respond to different measures included in the study. The participants were offered the possibility of getting to know the results once the study had ended.

### Results

#### Muslims

**FIGURE 3:**
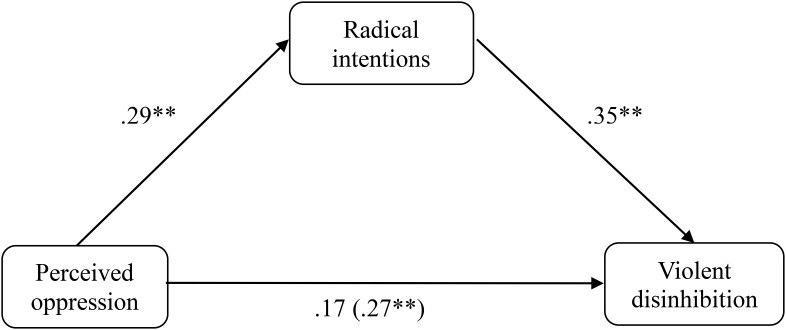
Mediation of oppression on violent disinhibition through the radical intentions in the Muslim sample of Study 2. ^∗^*p* < 0.05; ^∗∗^*p* < 0.01; ^∗∗∗^*p* < 0.001, *typified scores*.

As in Study 1, a mediation analysis was carried out to determine whether the radical intentions in the sample of Muslims showed a mediating effect on the relationship between perceived oppression and violent disinhibition (**Figure 3**). The results indicated the statistically significant predictive capacity of the perceived oppression on the radical intentions (β = 0.288, *p* = 0.004, *R*^2^ = 0.073), of the radical intention on violent disinhibition (β = 0.401, *p* < 0.001, *R*^2^ = 0.152), and of the perceived oppression on violent disinhibition (β = 0.269, *p* = 0.009, *R*^2^ = 0.062). Subsequently, a multiple linear regression was performed taking the perceived oppression and the radical intentions together as predictors, and the violent disinhibition as a criterion. The results indicated that when the radical intentions are introduced in this model, the perceived oppression loses its predictive capacity on violent disinhibition (β = 0.170, *p* = 0.089), whereas the radical intentions continue to show predictive capacity on the violent disinhibition (β = 0.354, *p* = 0.001). The Sobel (1982) test showed that there was a significant mediating effect of the radical intentions (*z* = 2.261, *p* = 0.024). The results obtained in the first study were corroborated once again; the radical intentions act as a mediator between perceived oppression and violent disinhibition in a sample of Muslims. Similar to Study 1, we replicated the analysis by changing the mediator variable for the dependent variable. The Sobel test showed a significant mediating effect of violent disinhibition on the predictive capacity of perceived oppression on radical intentions (*z* = 2.140, *p* = 0.032). In this case, it was a “feedback effect” (Kenny, 2014), which was not found in the first study.

**FIGURE 4:**
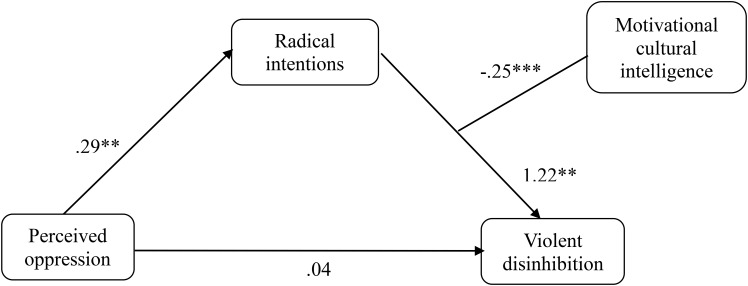
Moderated mediation of oppression on violent disinhibition through the radical intentions with motivational cultural intelligence as a moderator in the Muslim sample of Study 2. *^∗^p* < 0.05; ^∗∗^*p* < 0.01; ^∗∗∗^*p* < 0.001, *not* *typified scores*.

In a second step, analyses of moderate mediations were carried out by means of the macro PROCESS for SPSS (Hayes, 2013), with the objective to check whether the mediation of the radical intentions in the relationship between perceived oppression and violent disinhibition occurred independently of the components of cultural intelligence and cultural identity. To this end, the different components of cultural intelligence and cultural identity were used as moderators of the indirect effect (Models 7, 14, and 58 of Hayes were tested, in which moderation is found in both paths of the indirect effect). The only significant model appeared when the motivational component was a moderator in the relationship between the radical intentions and violent disinhibition (Model 14 of PROCESS, **Figure 4**). The results showed that the direct effect was not significant (*b* = 0.041, *SE* = 0.08, *p* = 0.489, 95% CI [−0.125; 0.207]), whereas the conditional indirect effect (*b* = −0.071; 95% CI [−0.151; −0.001]) occurred in those who had low (*b* = 0.097; 95% CI [0.002; 0.223]) and medium (*b* = 0.048; 95% CI [0.002; 0.148]) scores in motivational cultural intelligence, but not in those who scored high (*b* = −0.001; 95% CI [−0.038; 0.061]).

#### Non-Muslims

**FIGURE 5:**
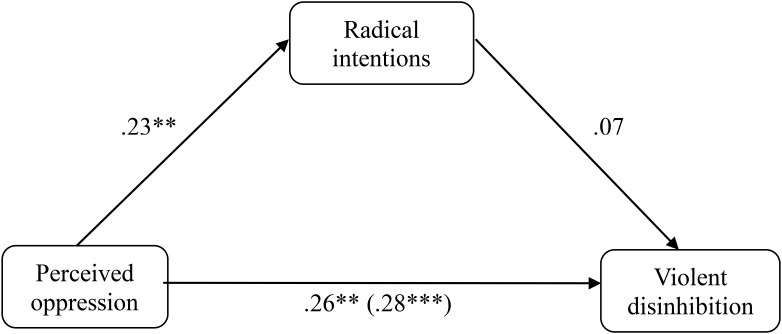
Mediation of oppression on violent disinhibition through the radical intentions in the non-Muslim sample of Study 2. *^∗^p* < 0.05; ^∗∗^*p* < 0.01; ^∗∗∗^*p* < 0.001, *typified scores*.

The same analyses were repeated with the sample of non-Muslims. In the first instance, the mediation of the radical intentions was analyzed (**Figure 5**). The results indicated that the perceived oppression had a predictive capacity on the radical intentions (β = 0.233; *p* = 0.003; *R*^2^ = 0.049) but not the radical intentions on violent disinhibition (β = 0.140; *p* = 0.071; *R*^2^ = 0.014). On the other hand, perceived oppression also had a predictive capacity on violent disinhibition (β = 0.281; *p* < 0.001; *R*^2^ = 0.073). Once these three simple regressions were carried out, a multiple linear regression was performed taking the perceived oppression and the radical intentions together as predictors and the violent disinhibition as a criterion. The results of this analysis indicated that when the radical intentions as a predictor are introduced into the multiple regression model, the perceived oppression maintains its predictive capacity on violent disinhibition (β = 0.264, *p* = 0.001), whereas the radical intentions continue without showing predictive capacity on violent disinhibition (β = 0.071, *p* = 0.366). Again, we examined the mediating effect after changing the mediator variable for the dependent variable. The Sobel test did not show a significant mediating effect from violent disinhibition (*z* = 0.878, *p* = 0.380).

**FIGURE 6:**
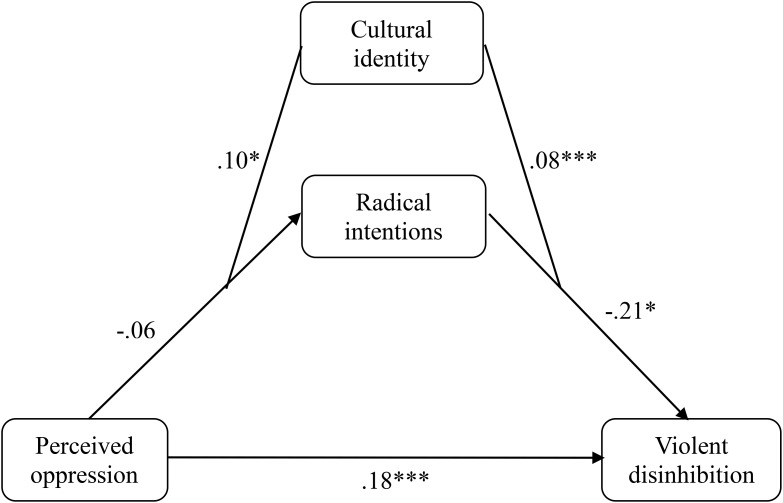
Moderated mediation of oppression on violent disinhibition through the radical intentions with cultural identity as a moderator in the non-Muslim sample of Study 2. *^∗^p* < 0.05; ^∗∗^*p* < 0.01; ^∗∗∗^*p* < 0.001, *not* *typified scores*.

After determining that mediation did not occur, unlike with the sample of Muslims, the same analyses of moderate mediations were carried out with the sample of Muslims to estimate whether the introduction of the components of cultural intelligence or cultural identity as moderators would produce the indirect effect. It was found that identification with the Western-Christian culture was a significant moderator of the indirect effect, but not the cultural intelligence. That is, Western-Christian cultural identity moderated the relationship between oppression and the radical intentions while moderating the relationship between the radical intentions and violent disinhibition (Model 58, **Figure 6**). The results showed that the direct effect was significant (*b* = 0.180, *SE* = 0.05, *p* < 0.001, 95% CI [0.074; 0.287]) whereas the conditional indirect effect occurred for those with high scores (*b* = 0. 067; 95% CI [0.009; 0.154]) but not for those with medium (*b* = 0.006; 95% CI [−0.012; 0.044]) or low (*b* = −0.007; 95% CI [−0.045; 0.023]) scores.

### Discussion

The hypothesis proposed in the first study was confirmed in this study. Although there was mediation by the radical intentions in the relationship between oppression and violent disinhibition in the Muslim sample, in the non-Muslim sample, no such mediation was found, although it was found that oppression was a significant predictor of the radical intentions. On the other hand, several moderators of the proposed mediations were found in both samples. In the case of Muslims, the motivational component of cultural intelligence moderated the relationship between the radical intentions and violent disinhibition. Specifically, mediation only occurred when participants had low or medium cultural motivation. Conversely, in the non-Muslim sample, it was only when the participants showed high identification with the Western-Christian culture that the mediation of the radical intentions in the relationship between oppression and violent disinhibition took place.

## General Discussion

Violent radicalization is an issue causing great concern in Western countries. The return of radicalized people who have fought in countries of the Middle East is a new problem that Western societies must face (Barrett, 2017; Reinares and García-Calvo, 2017; Europol, 2018). Specifically, the possibility that these returnees act like recruiters increases the risk of radicalization. To make matters worse, several factors have been proposed in favor of violent radicalization; among them are relative deprivation, the need for cognitive closure, social exclusion, or perceived oppression (Trujillo et al., 2006a,b). Our studies focus on determining whether the quest for significance can encourage people to use violence once they have been subjected to a violent narrative. It was also considered that the influence of these factors would be different depending on whether the groups were Muslims or non-Muslims. Consequently, the studies propose to evaluate in Muslims and non-Muslims if the perceived oppression was a predictor of the violent disinhibition that acted through the radical intentions. Both studies confirmed this hypothesis in each of the Muslim samples. In contrast, in the non-Muslim sample, oppression turned out to be a predictor of violent disinhibition, but the indirect effect of the radical intentions was not found. Another point of discussion is the feedback effect that appeared in the Muslim sample in the second study. The significant prediction of changing the position of the mediator variable and dependent variable suggests that the two influence each other. Therefore, we should be cautious with the interpretations when this effect was not replicated. Additional studies, including the manipulation of the mediator variable, are needed to establish a conclusion.

In addition, it was believed that the influence of the perceived oppression on violent disinhibition through the radical intentions would depend on other variables, more precisely cultural-religious identity and cultural intelligence. It was found that cultural intelligence, specifically the motivational component, moderated the relationship between the radical intentions and violent disinhibition in the sample of Muslims. That is, the indirect effect of the radical intentions on violent disinhibition occurred only in those Muslims with a lower level of motivational cultural intelligence. Specifically, because motivational cultural intelligence is defined as an intrinsic interest in experiencing other cultures and interacting with different people (Ang and Van Dyne, 2008), those with less interest are more prone to show a violent disinhibition. On the other hand, in the sample of non-Muslims, it was found that a high identification with the Western-Christian culture allowed the indirect effect of radical intentions on the relationship between perceived oppression and violent disinhibition.

According to the significance quest theory (Kruglanski et al., 2009a, 2013, 2014, 2017), the results obtained are consistent with previous studies, where it has been determined that the loss of significance (the perceived oppression in this case) can predict violent disinhibition or extremism (Webber et al., 2018). In addition, the radical intentions, understood as the willingness to carry out illegal and violent activities (Moskalenko and McCauley, 2009), act as a mediator of this relationship. Therefore, these studies add another variable that would act as a mediator in the significance quest theory. On the other hand, from the perspective of the psychosocial model of recruitment, the intention of radicalization contributes to a greater vulnerability for Muslims. In other words, during the phases of disinhibition and violent legitimation, the new radical will end up considering violent actions as lawful and instrumentally valid to achieve his objectives, which will conform to those imposed by his group. Sometimes, given the right conditions, people who are well indoctrinated and radicalized in violence may end up firmly believing that death for killing is necessary to give the maximum possible meaning to his existence and to be able to enjoy a pure life beyond the mundane. In this way, violent disinhibition will allow the individual to perceive violent actions as lawful and instrumentally valid in achieving the group’s objective (Trujillo and Moyano, 2018). In addition, when violent narrative induces the violent disinhibition, interpreting this as a possible mental illness loses its validity. This option seems more plausible when violent disinhibition is triggered by the perception of oppression. However, these arguments are only speculations.

Regarding the moderating variables, cultural identity and cultural intelligence, both factors of vulnerability (or protection, depending on their degree) within the psychosocial model of recruitment, it can be said that they complement the contributions of the significance quest theory. In the case of Muslims, cultural intelligence seems to be a factor associated with a better integration and understanding of the Western-Christian culture, but it is not relevant for non-Muslims in this context. Consequently, it can be argued that this result could be related to a lack of integration; those who are less interested in mainstream culture are more likely to use violence. Likewise, cultural identity is of greater importance for non-Muslims, probably because Muslims have a stronger identification with their culture and religion, which can create a glass ceiling effect. In future studies, it is essential to verify the relationship that these variables could have with the need for cognitive closure, which has been seen as a mediator of the relationship between the loss of significance and extremism (Webber et al., 2018).

Taking into account the results as a whole, although more empirical evidence is needed, the effects found are consistent with the reviewed theoretical models and previous research. However, it is necessary to highlight some of the limitations of this study. The first limitation lies in the structure of the studies. The questionnaire designs used do not allow us to determine the direction of the found effects; therefore, experimental studies that could validate the effects of the proposed factors contributing to the radicalization, as well as the moderating effect of the cultural identity and cultural intelligence, would be necessary (although experimental studies on this subject will also carry their own set of pitfalls). Another limitation is the difficulty in interpreting why the other components of cultural intelligence did not have the same effects as the motivational component, for which it would be necessary to carry out more studies and with different types of samples. Finally, we highlight that people of different contexts were grouped by their religious identification (Study 1) or religious-cultural identification (Study 2). This could be considered a limitation given that other characteristics or identities could be more important. On the other hand, in making them choose a religion, their religious identity was activated; this may explain the reason for the replication. We recommend that future studies analyze the possible cultural differences that could influence the radicalization processes.

In summary, these studies offer a contribution to an underexplored field: the moderating effect of cultural intelligence and its respective subdimensions on violent disinhibition. They also provide more empirical data on the importance of group identity, perceived oppression, and radical intentions, which could add new pieces to the puzzle that radicalization presents. This research provides new evidence and an explanation to the different pathways to radicalization of Muslims and non-Muslims in the context of Western societies. The loss of significance seems to be a trigger for violent radicalization that has a higher impact on Muslims, and this could be potentially explained by the socioeconomic marginalization they face or perceive. The conjunction of the loss of significance and radical intentions seems to exacerbate the likelihood of a process of violent disinhibition, which, in turn, increases the vulnerability vis-à-vis potential recruiters and the risk of engaging in terrorist activity.

## Ethics Statement

This study was carried out in accordance with the guidelines stated by the Vice-rectory of Research and Scientific Policy of the University of Granada, fulfilling the requirements of informed consent and data protection stated by the Spanish Organic Law 15/1999. The protocol was approved by the Ethics Committee for Research of the University of Granada (No 170/CEIH/2016). This study was conducted by using questionnaires. Hence, all participants were informed in writing about the objectives of the study, and signed their consent to voluntarily participate in the study. Once the study was concluded, we provided feedback to all respondents regarding the research findings.

## Author Contributions

RL and MaM: development of field research, preparation and analysis of data, writing research reports, coordination with institutions, and preparation of events and consulting, among other functions. MiM and HT: project management, data analysis, and writing of the research report.

## Conflict of Interest Statement

The authors declare that the research was conducted in the absence of any commercial or financial relationships that could be construed as a potential conflict of interest.
